# Supplementary material for: Design of a Tribotester Based on Non-Contact Displacement Measurements
Source: Micromachines (Basel). 2019 Oct 31;10(11):748. doi: 10.3390/mi10110748 (PMC6915551; doi:10.3390/mi10110748)
Supplement: Supplementary file 1 [file micromachines-10-00748-s001.zip › Supplementary Materials-layout.pdf]

micromachines

Supporting information

Design of a Tribotester based on Non-Contact Displacement Measurements

Chang-Lae Kim and Yoon-Gyung Sung*

Department of Mechanical Engineering, Chosun University, Gwangju 61452, Korea; kimcl@chosun.ac.kr
* Correspondence: sungyg@chosun.ac.kr; Tel.: +82-62-230-7181

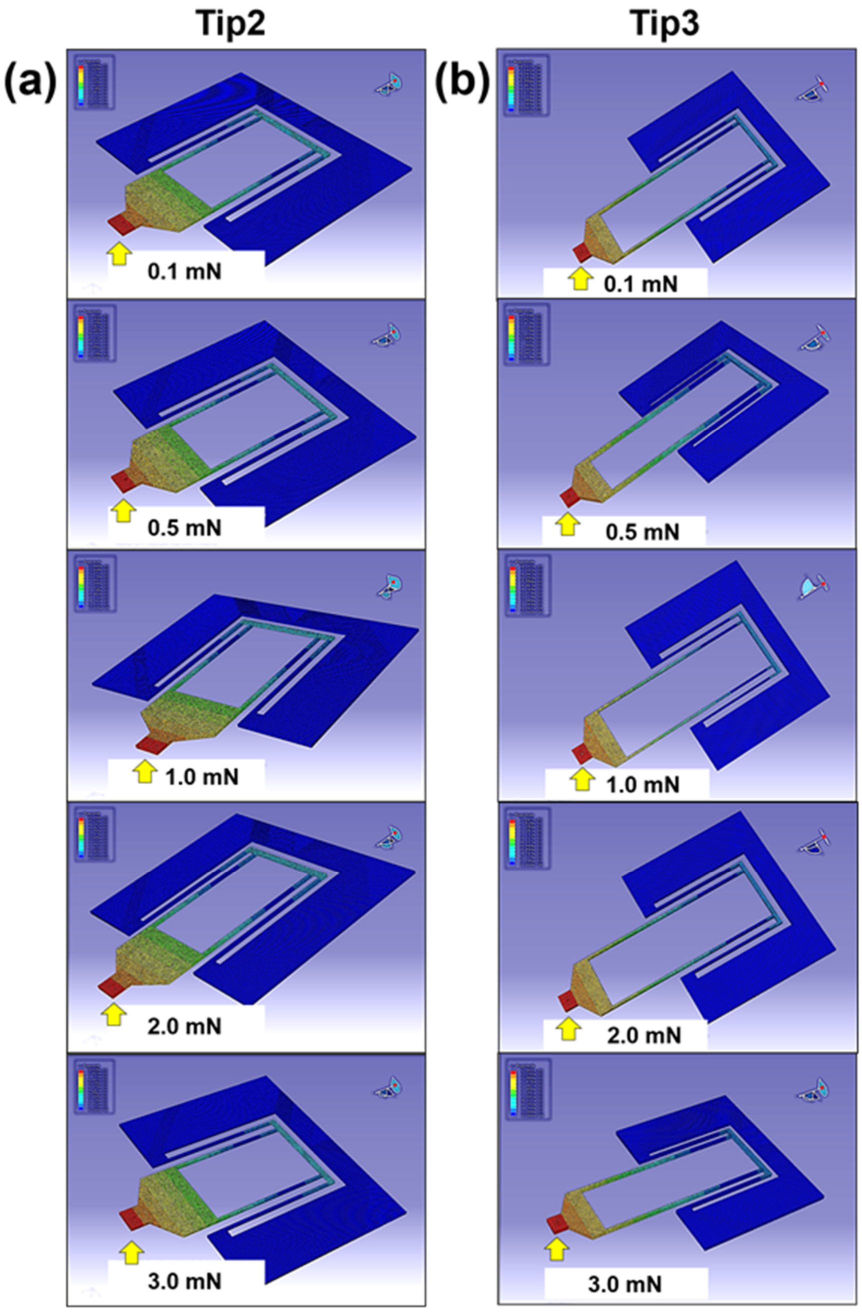

Figure S1. Displacement results for (**a**) Tip 2 and (**b**) Tip 3 depending on the normal forces (0.1, 0.5, 1.0, 2.0, and 3.0 mN) based on FEA simulations.

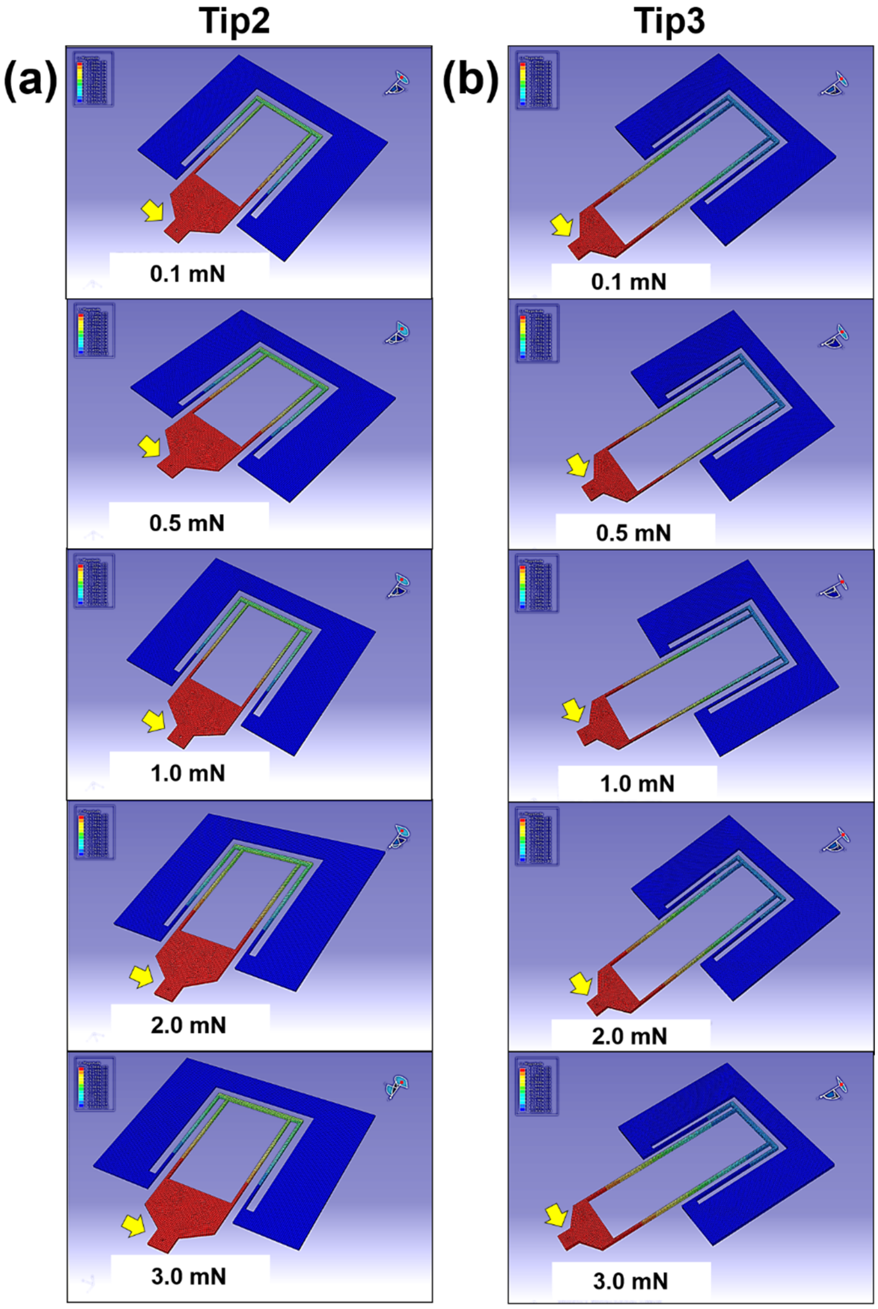

Figure S2. Displacement results for (**a**) Tip 2 and (**b**) Tip 3 depending on the friction forces (0.1, 0.5, 1.0, 2.0, and 3.0 mN) based on FEA simulations.

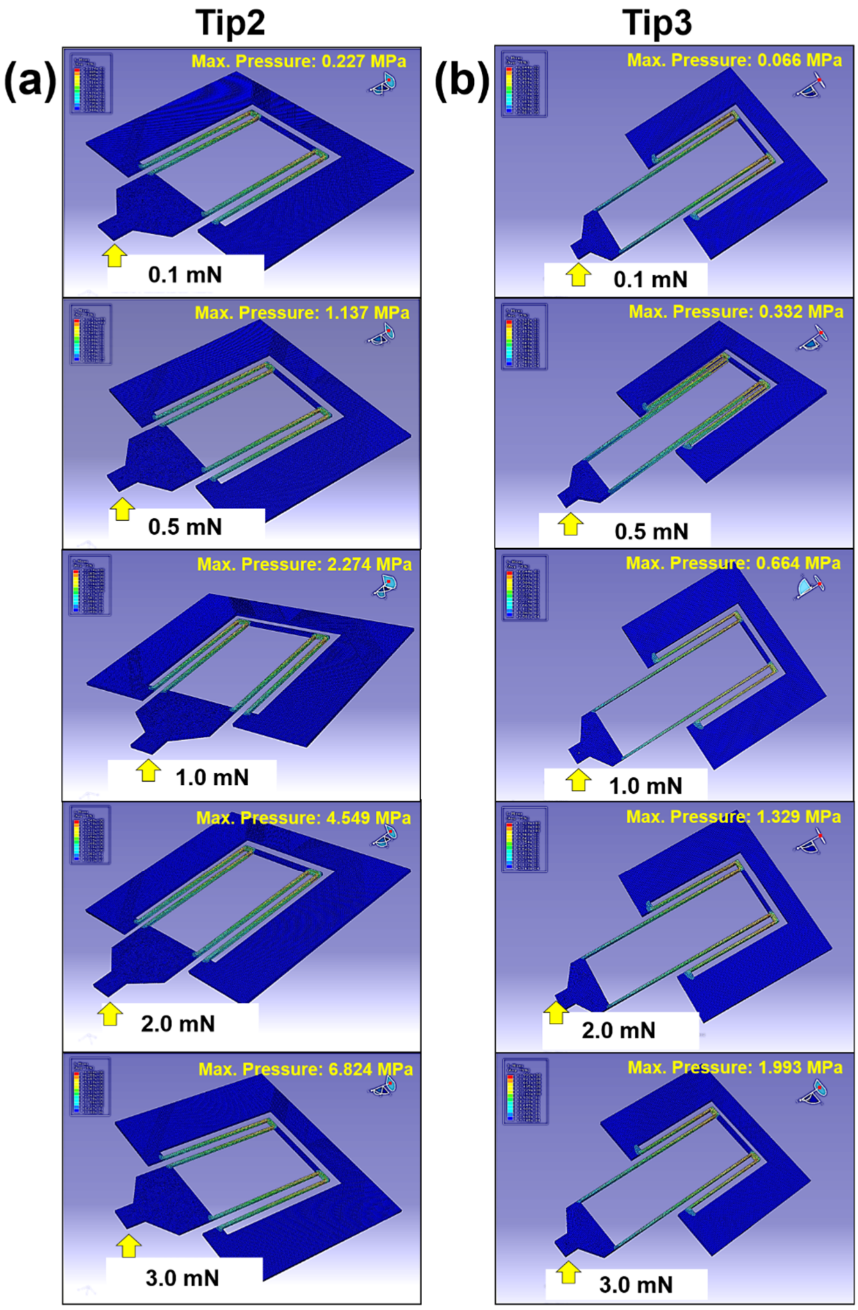

Figure S3. Pressure results for (**a**) Tip 2 and (**b**) Tip 3 depending on the normal forces (0.1, 0.5, 1.0, 2.0, and 3.0 mN) based on FEA simulations.

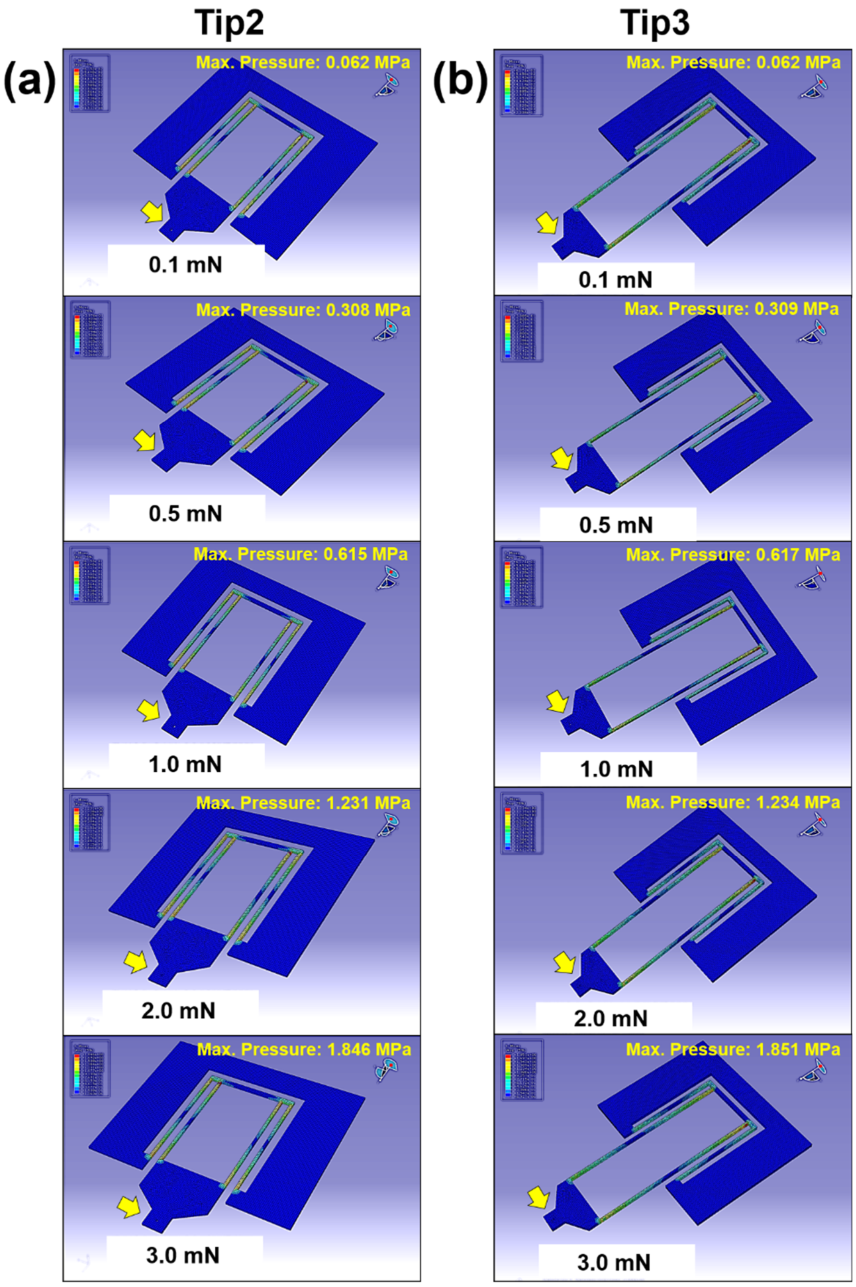

Figure S4. Pressure results for (**a**) Tip 2 and (**b**) Tip 3 depending on the friction forces (0.1, 0.5, 1.0, 2.0, and 3.0 mN) based on FEA simulations.

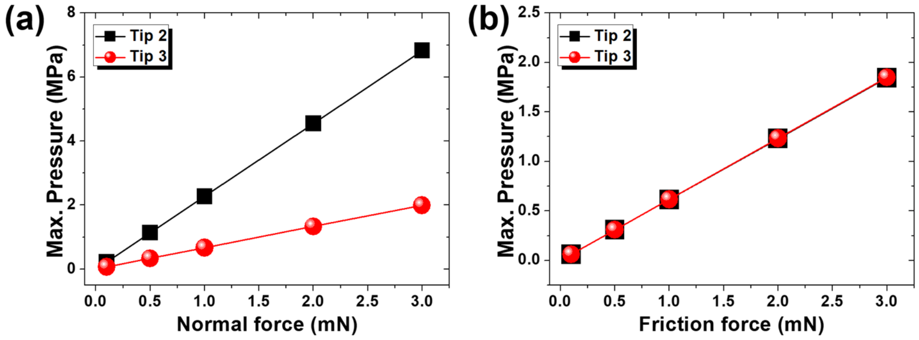

Figure S5. Pressure variations of Tip 2 and Tip 3 depending on the (**a**) normal forces and (**b**) friction forces, respectively (0.1, 0.5, 1.0, 2.0, and 3.0 mN).

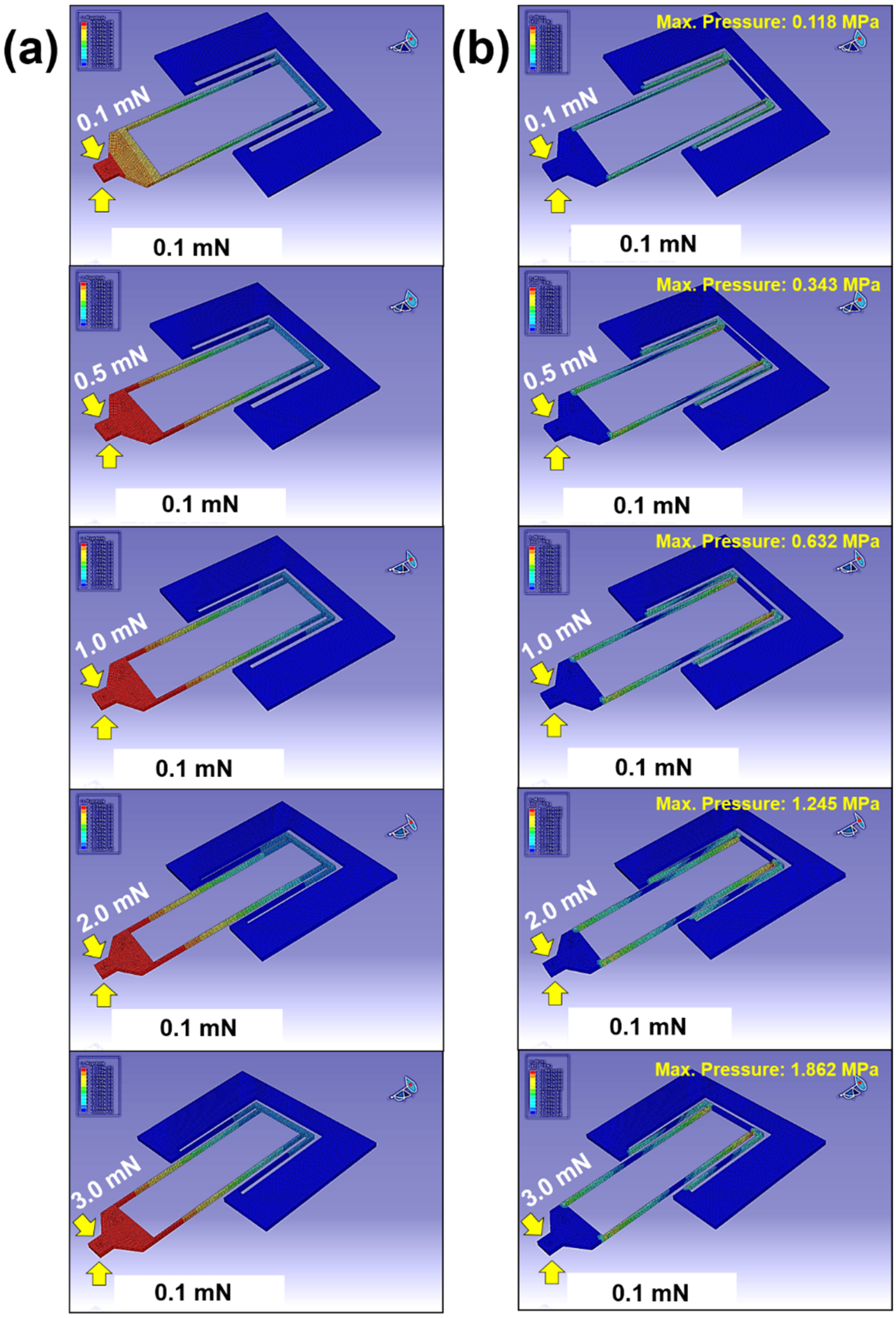

Figure S6. (**a**) Displacement and (**b**) pressure changes of Tip 3 depending on the friction force under a normal force of 0.1 mN.

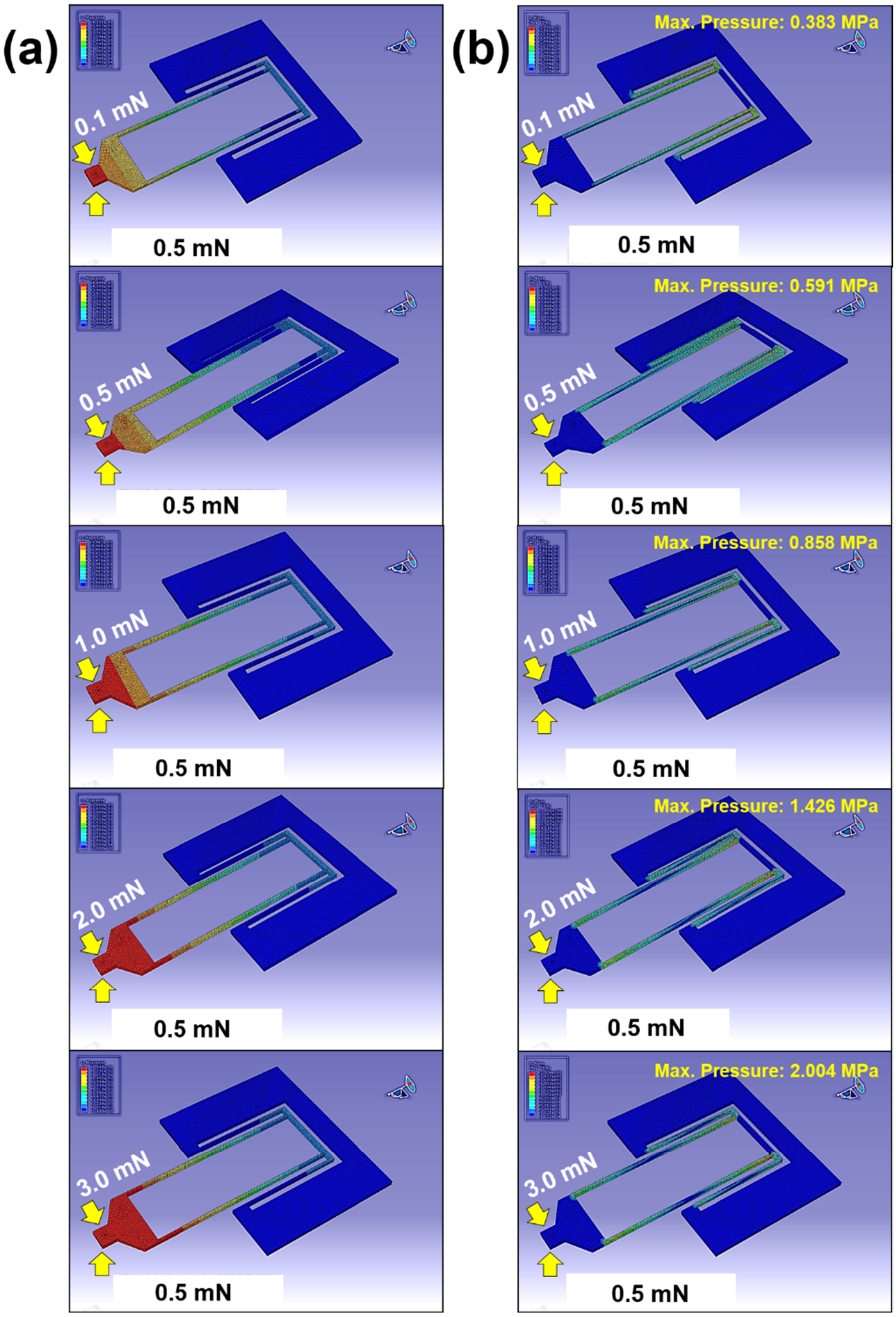

Figure S7. (**a**) Displacement and (**b**) pressure changes of Tip 3 depending on the friction force under a normal force of 0.5 mN.

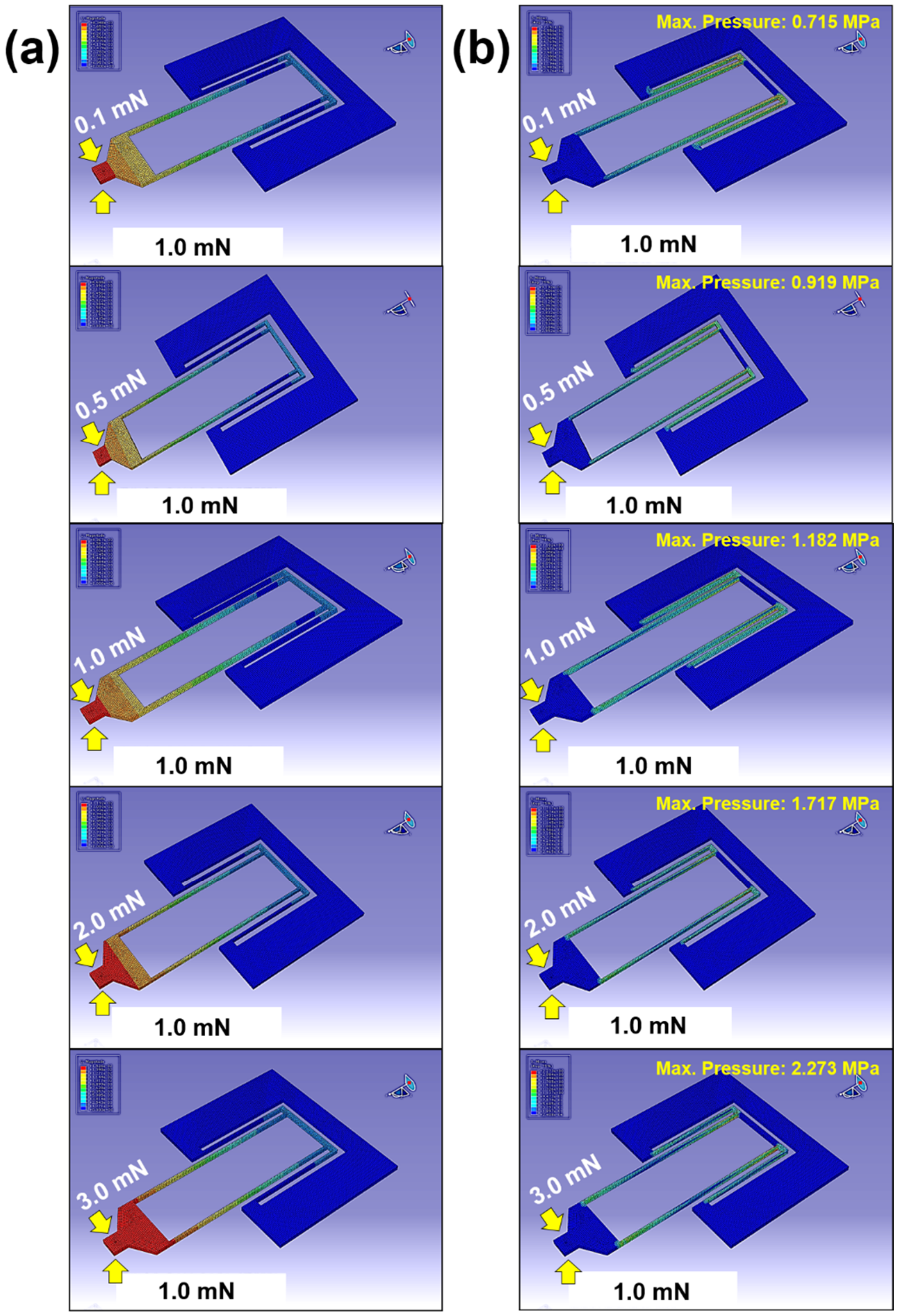

Figure S8. (**a**) Displacement and (**b**) pressure changes of Tip 3 depending on the friction force under a normal force of 1.0 mN.

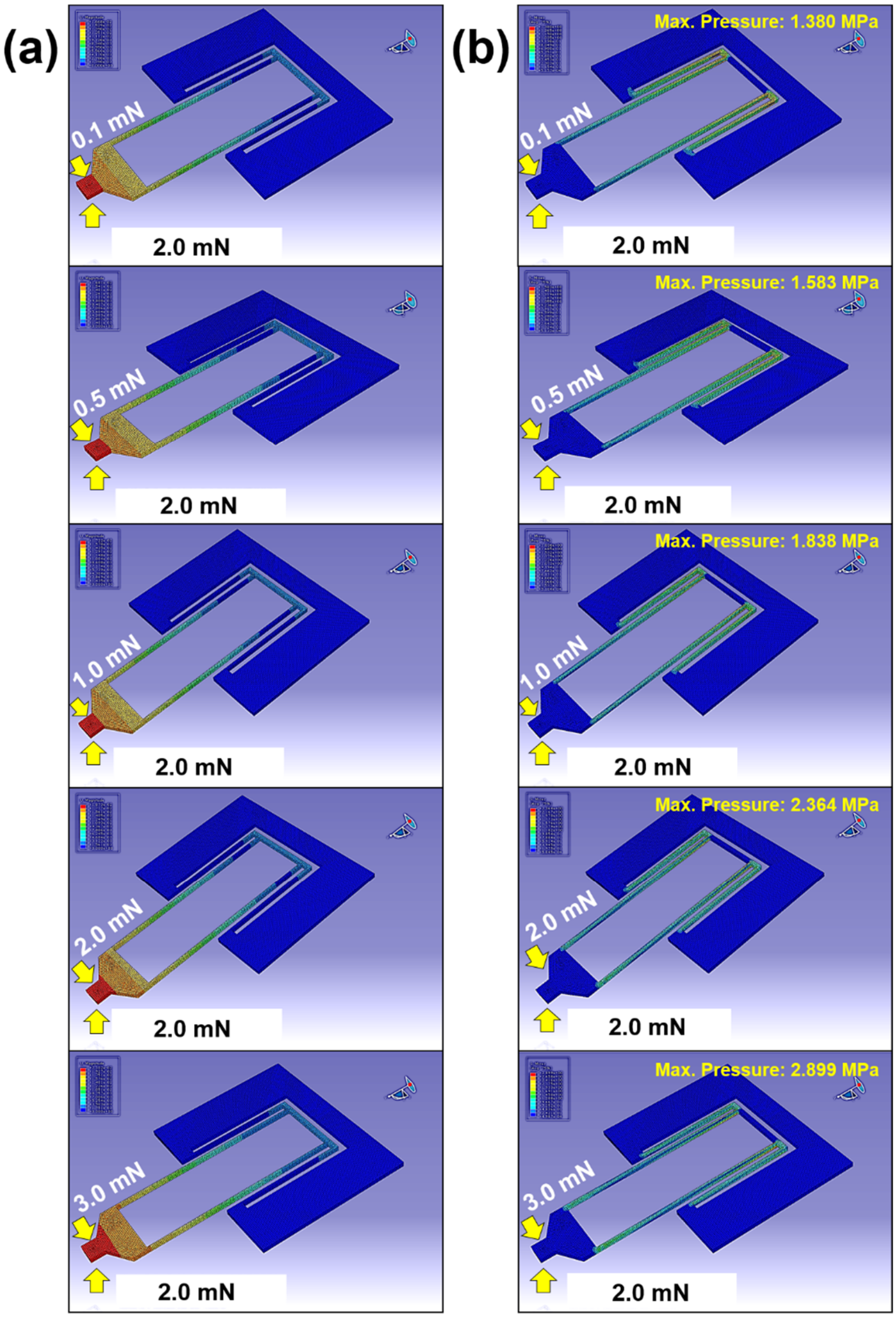

Figure S9. (**a**) Displacement and (**b**) pressure changes of Tip 3 depending on the friction force under a normal force of 2.0 mN.

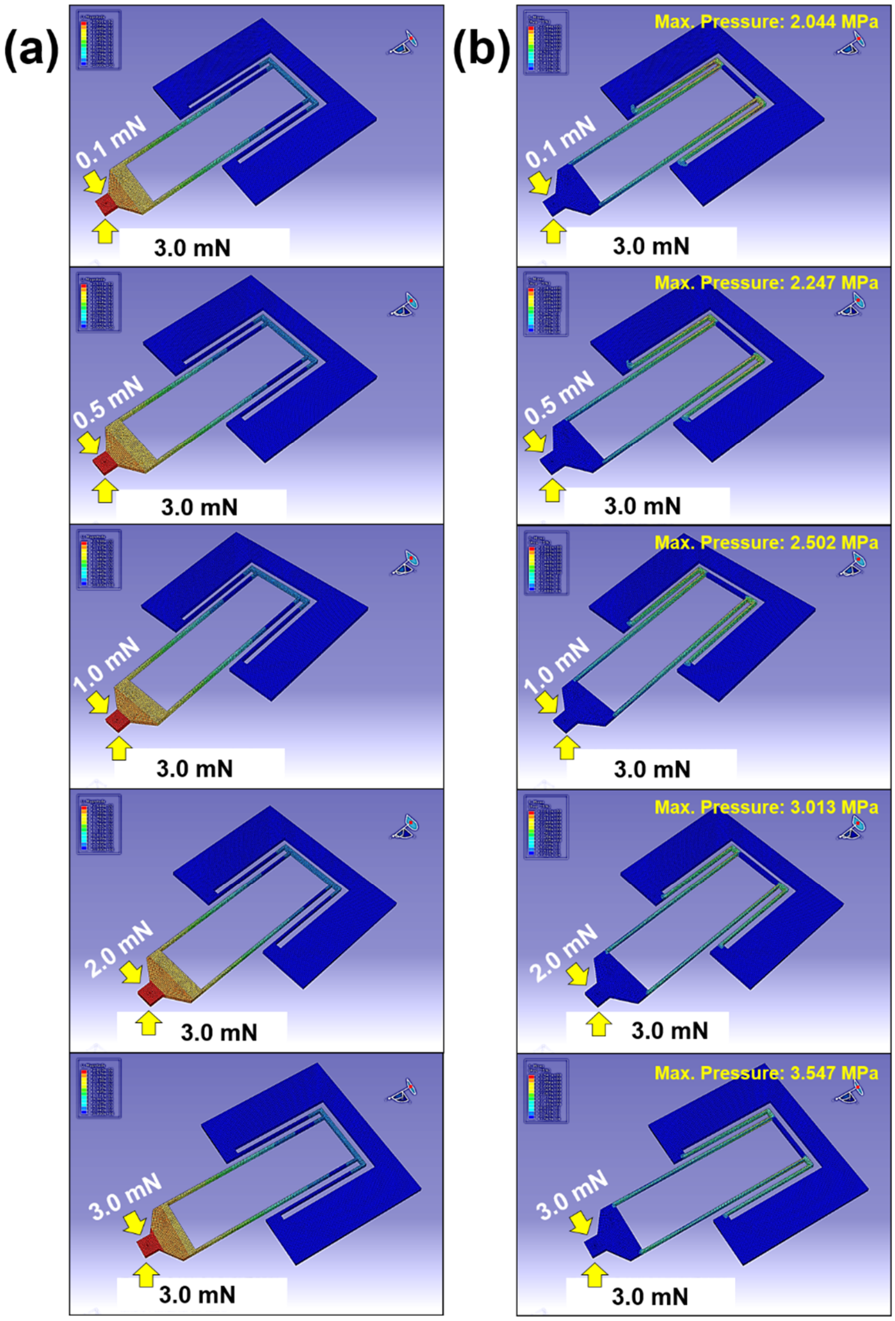

Figure S10. (**a**) Displacement and (**b**) pressure changes of Tip 3 depending on the friction force under a normal force of 3.0 mN.

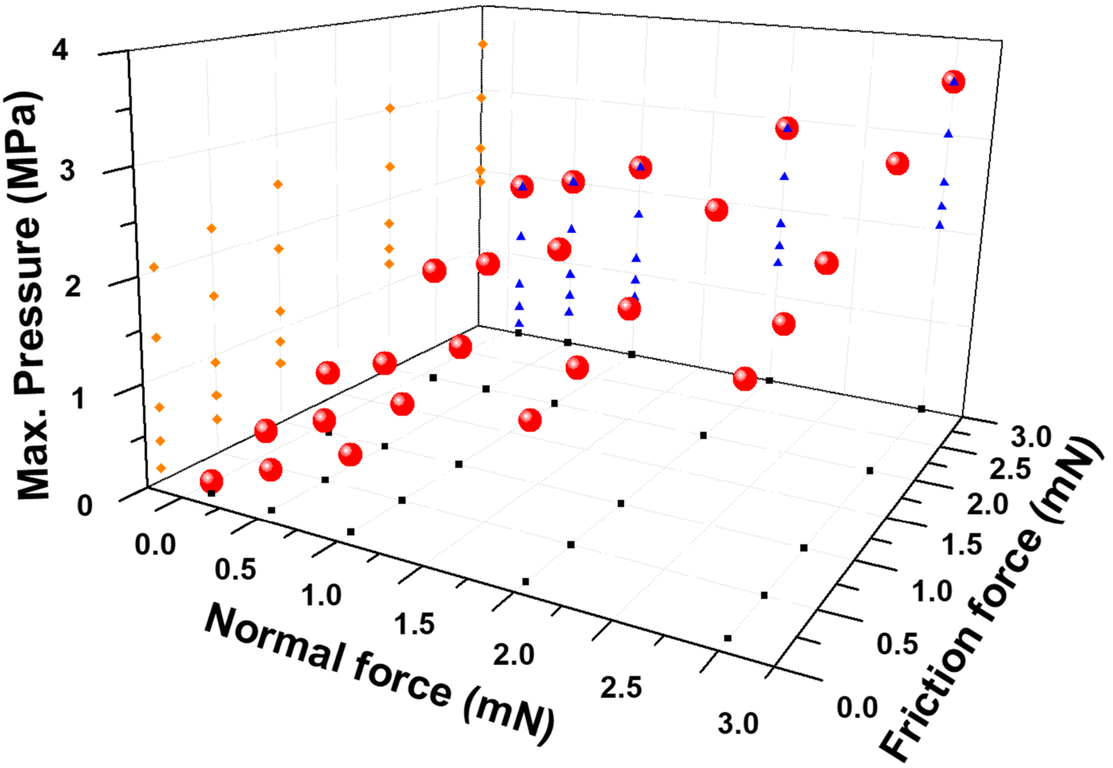

Figure S11. Relationship between the maximum pressure and normal/friction forces generated at the edge parts of the arms of the cantilever tip.
