# Supplementary figures and images for: Design of a Tribotester Based on Non-Contact Displacement Measurements
Source: Micromachines (Basel). 2019 Oct 31;10(11):748. doi: 10.3390/mi10110748 (PMC6915551; doi:10.3390/mi10110748)

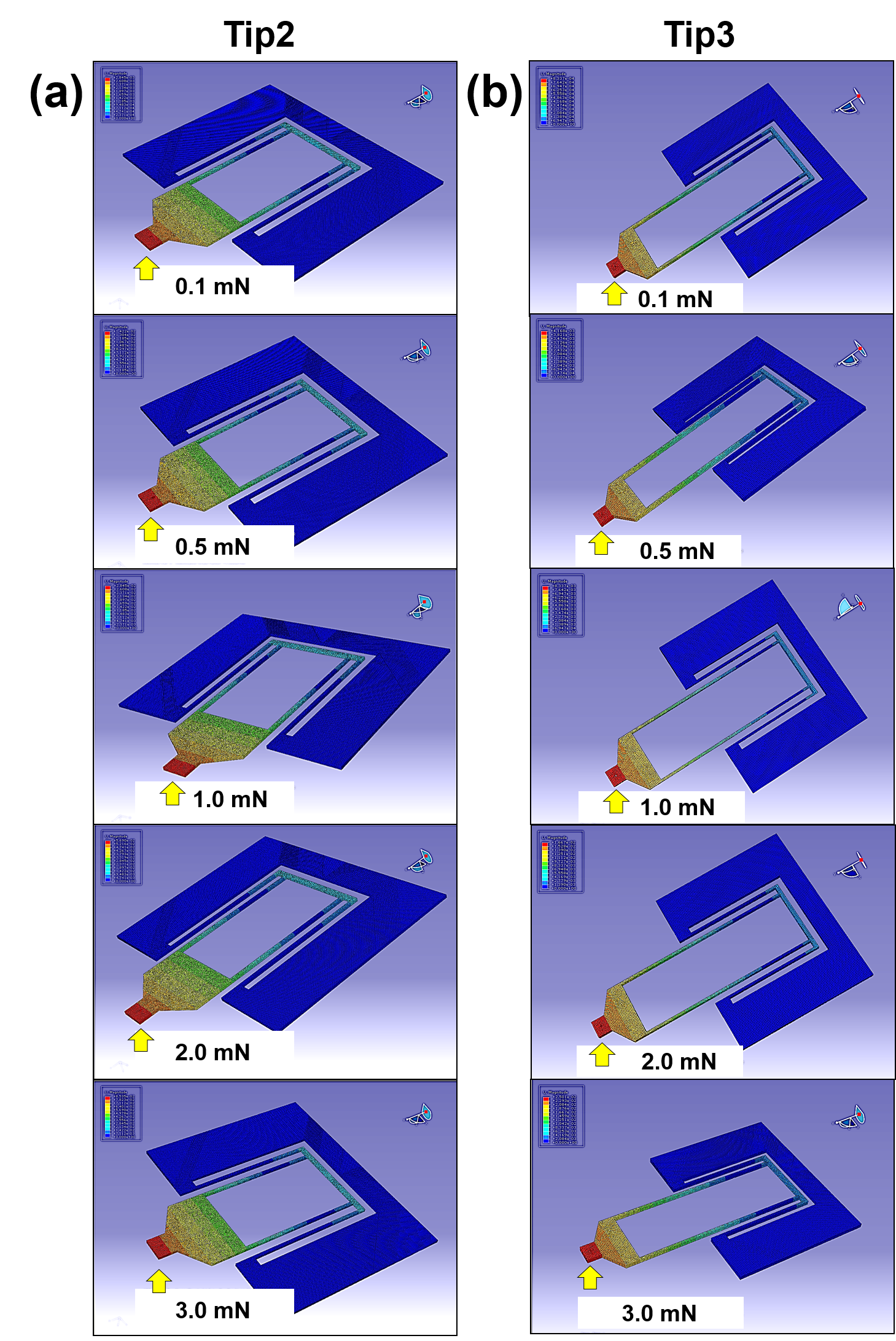

Supplement: Supplementary file 1 [file micromachines-10-00748-s001.zip › Figure S1.tiff]

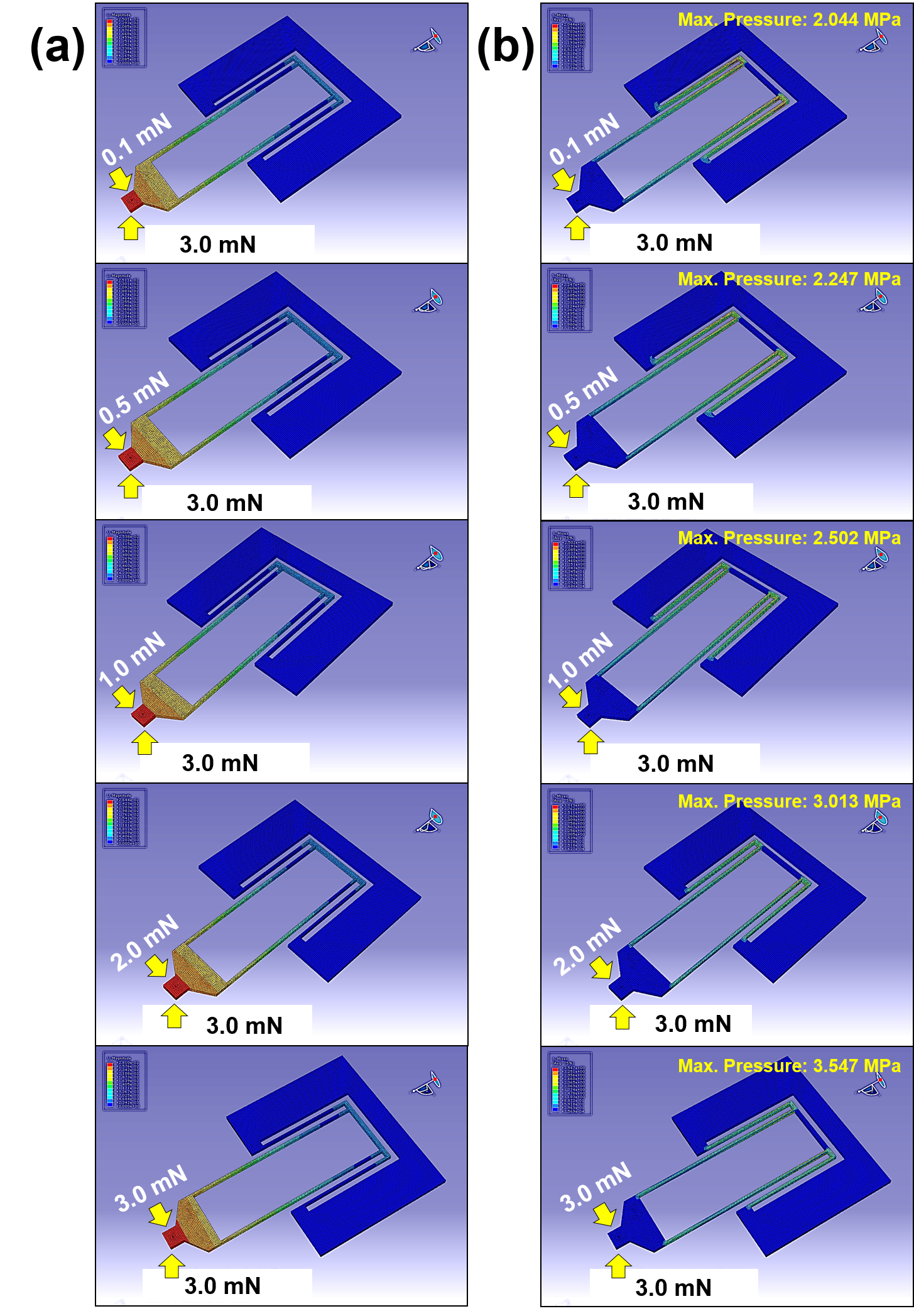

Supplement: Supplementary file 1 [file micromachines-10-00748-s001.zip › Figure S10.tiff]

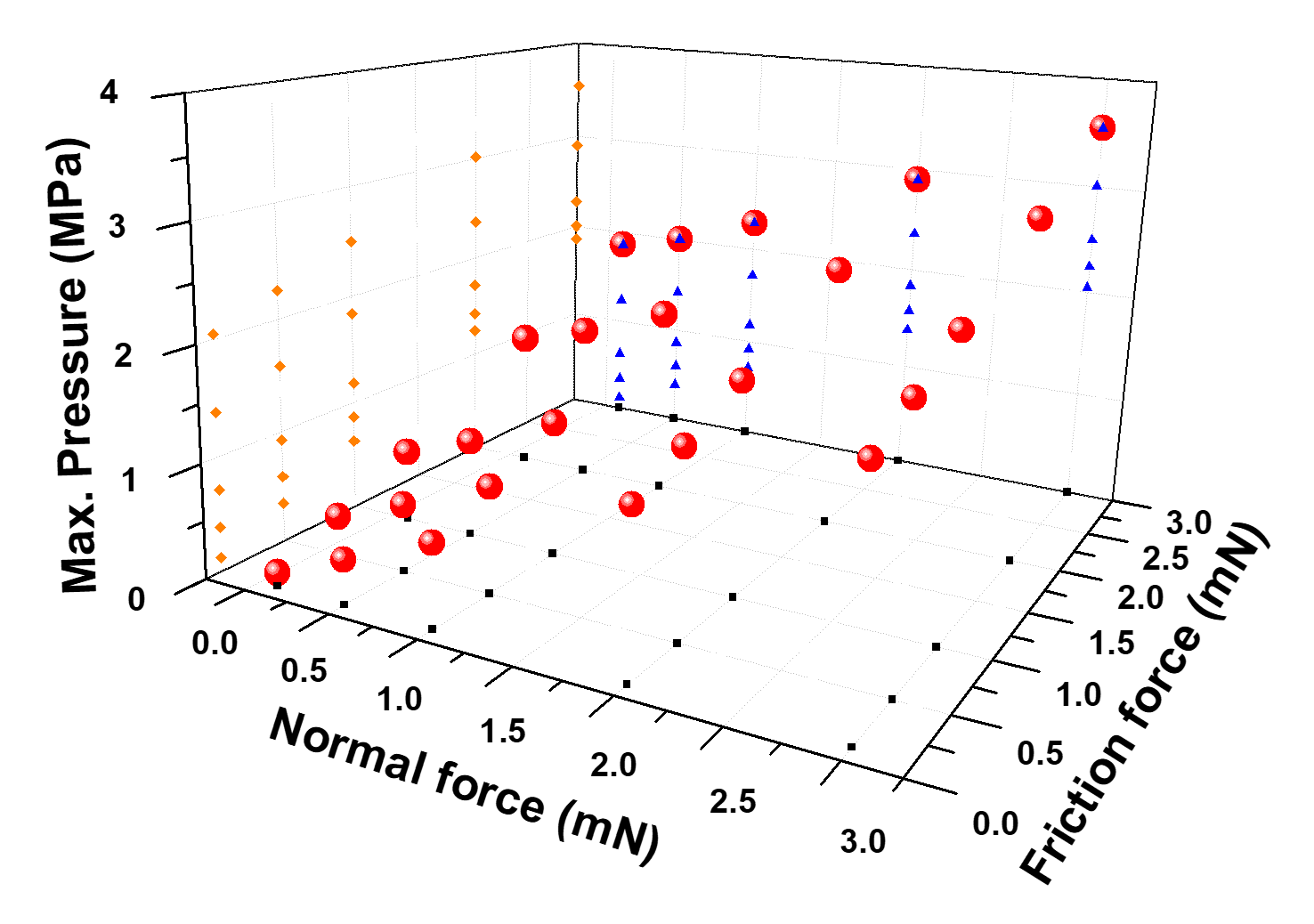

Supplement: Supplementary file 1 [file micromachines-10-00748-s001.zip › Figure S11.tiff]

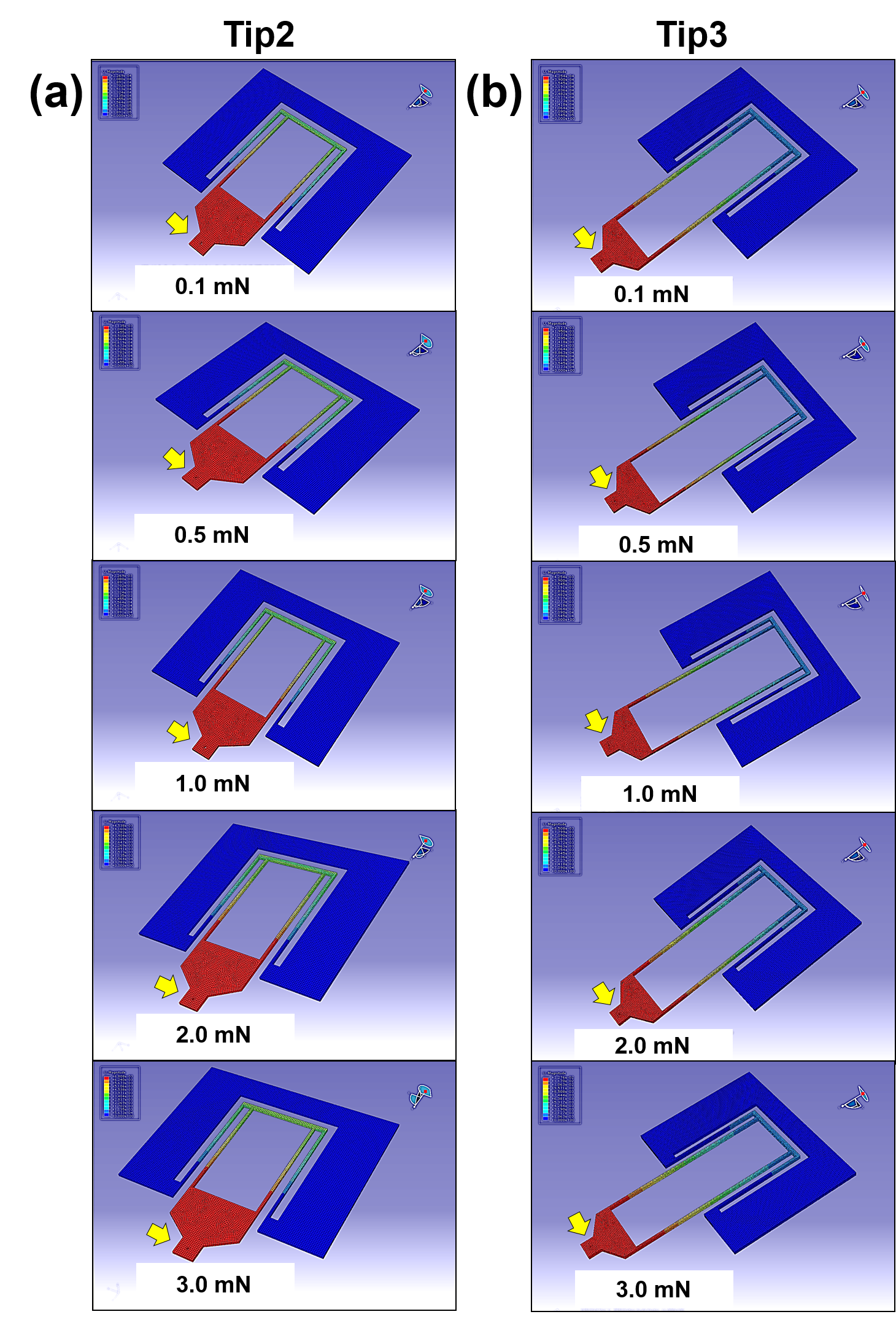

Supplement: Supplementary file 1 [file micromachines-10-00748-s001.zip › Figure S2.tiff]

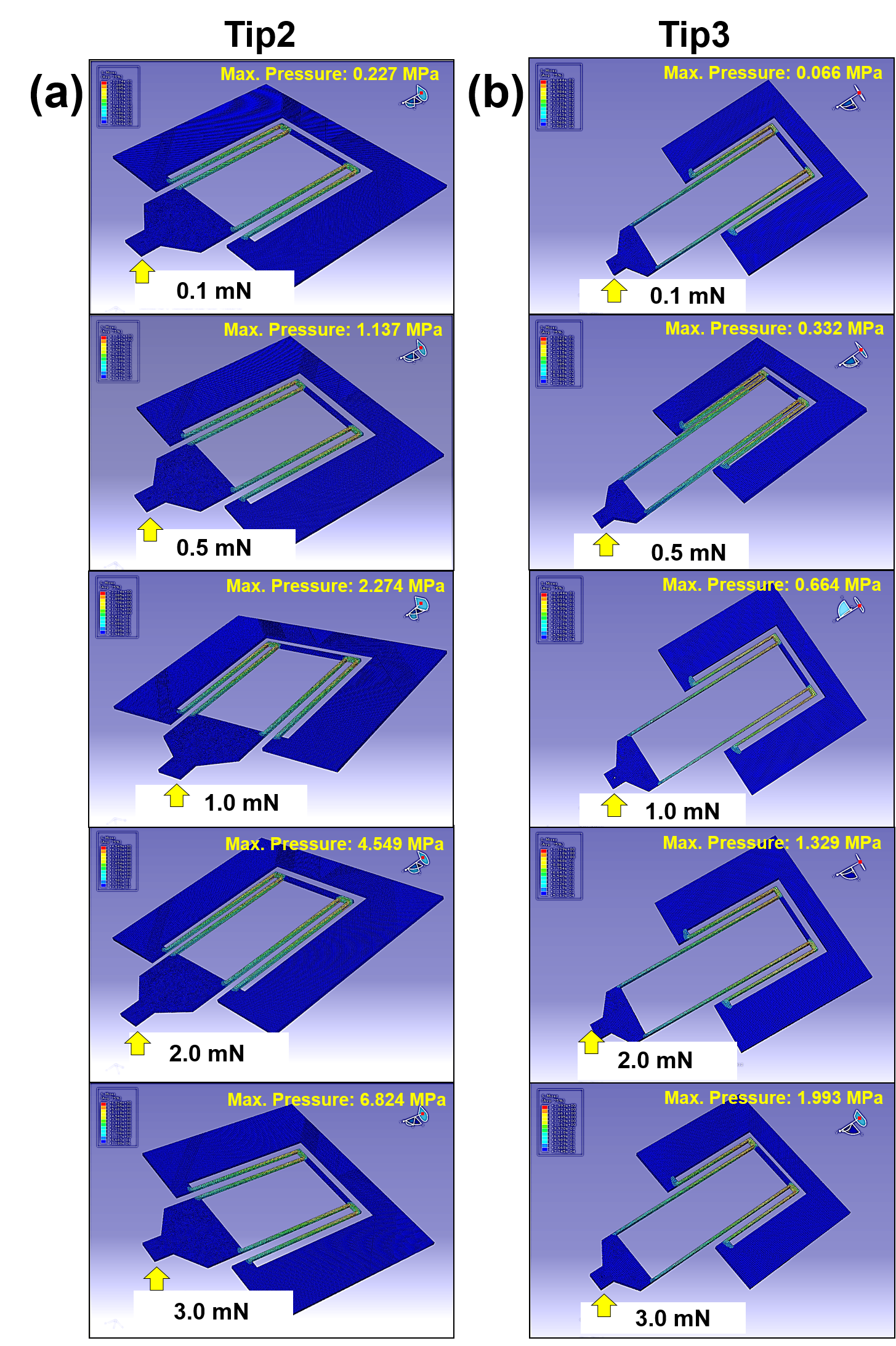

Supplement: Supplementary file 1 [file micromachines-10-00748-s001.zip › Figure S3.tiff]

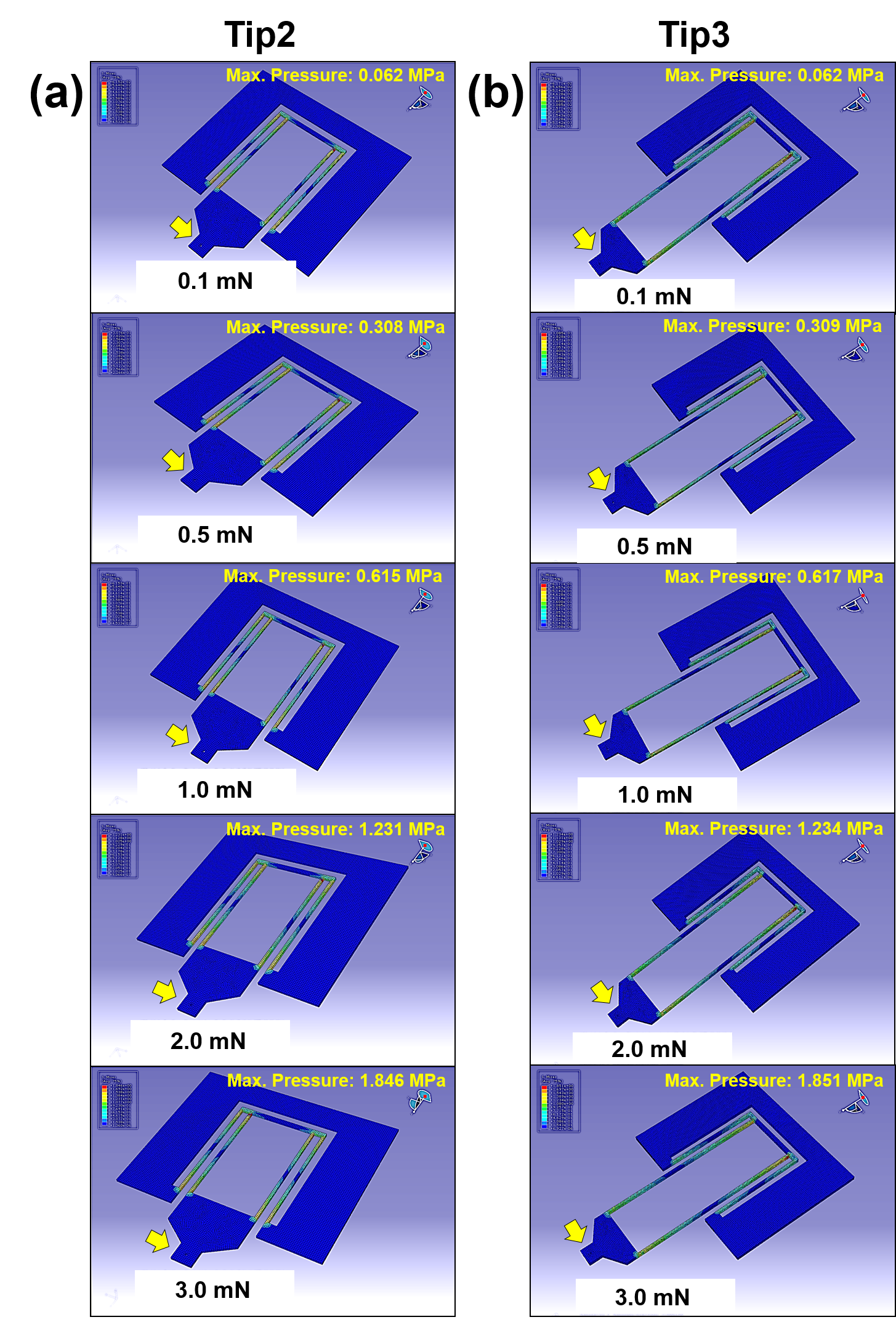

Supplement: Supplementary file 1 [file micromachines-10-00748-s001.zip › Figure S4.tiff]

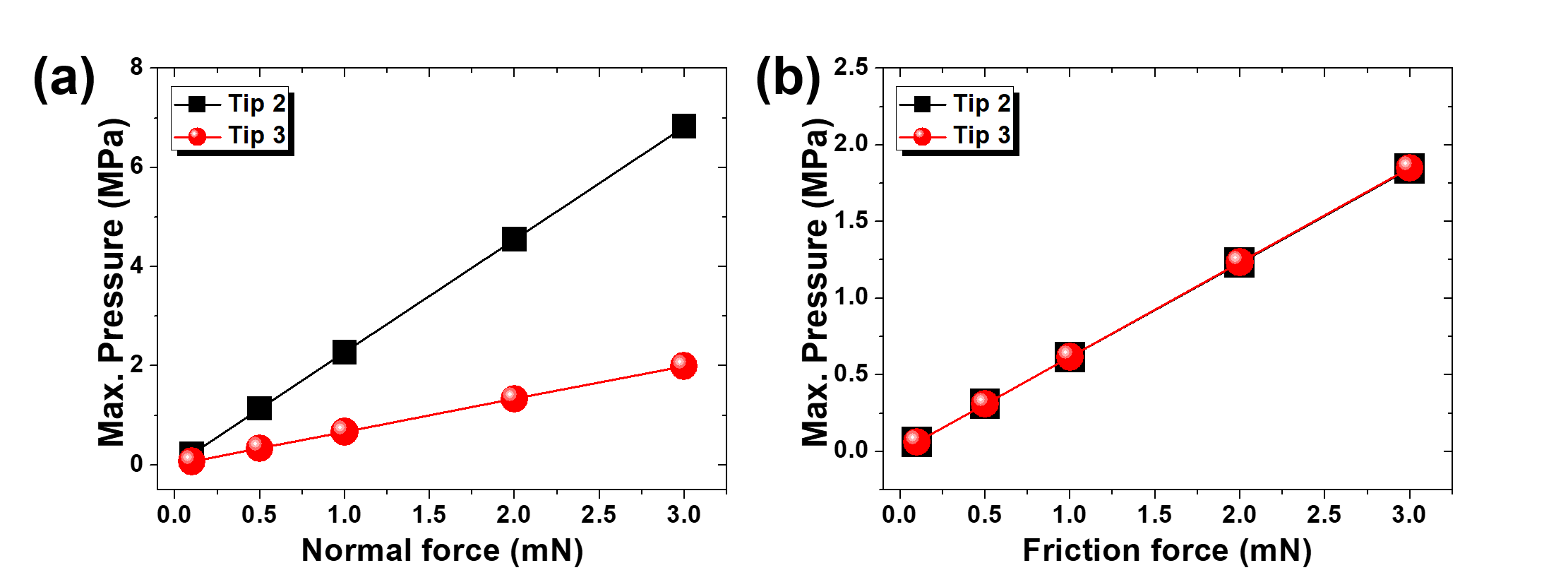

Supplement: Supplementary file 1 [file micromachines-10-00748-s001.zip › Figure S5.tiff]

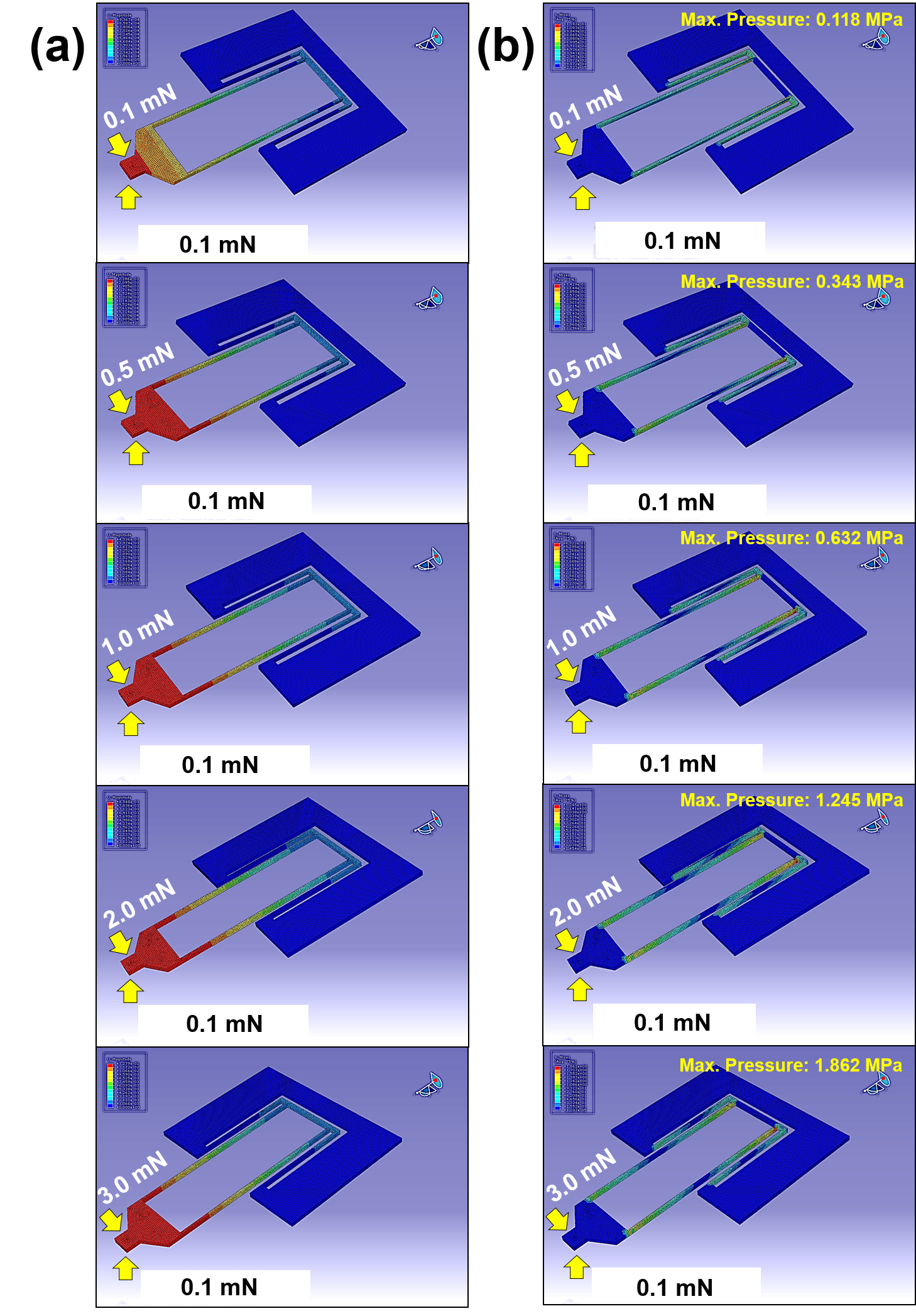

Supplement: Supplementary file 1 [file micromachines-10-00748-s001.zip › Figure S6.tiff]

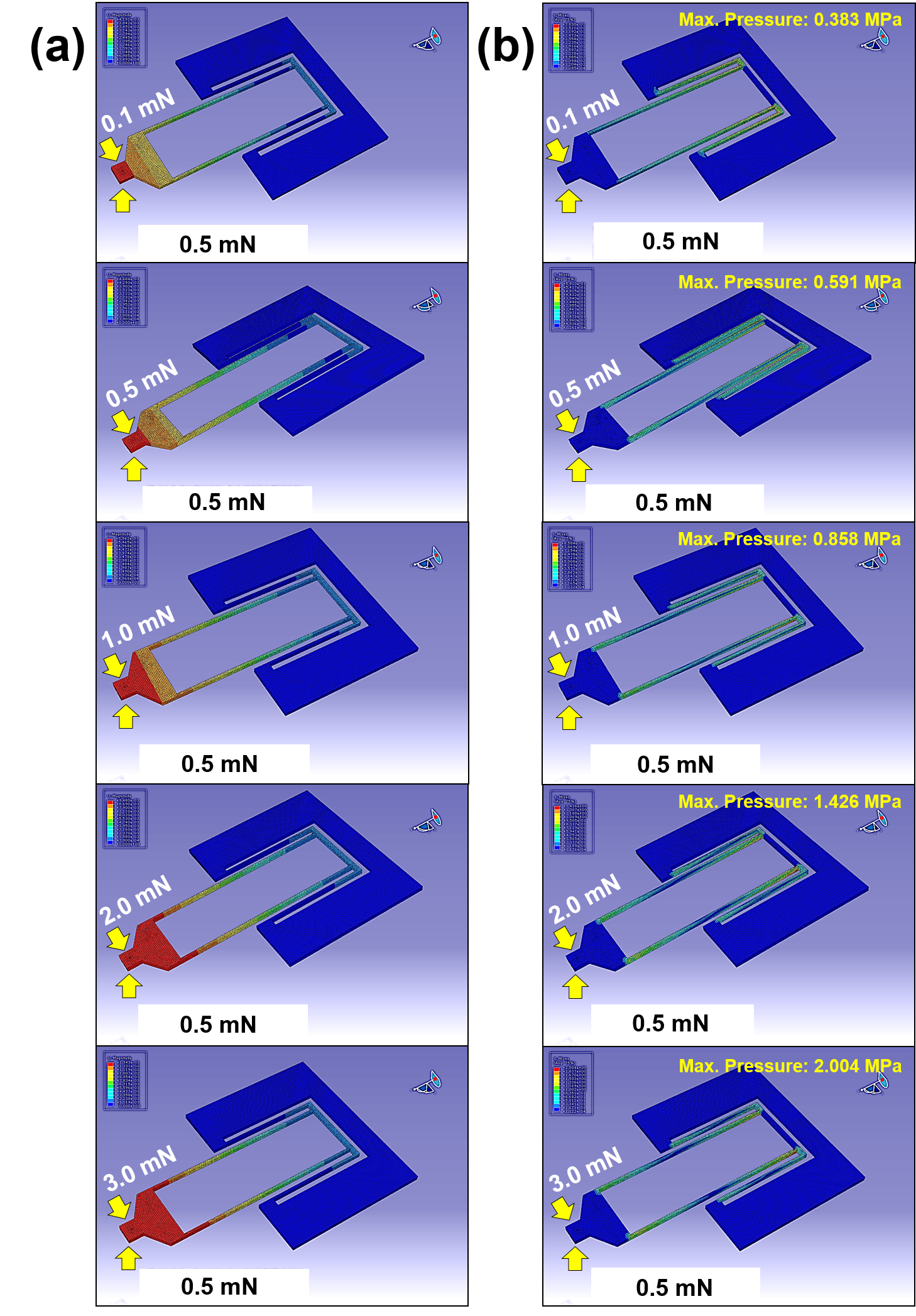

Supplement: Supplementary file 1 [file micromachines-10-00748-s001.zip › Figure S7.tiff]

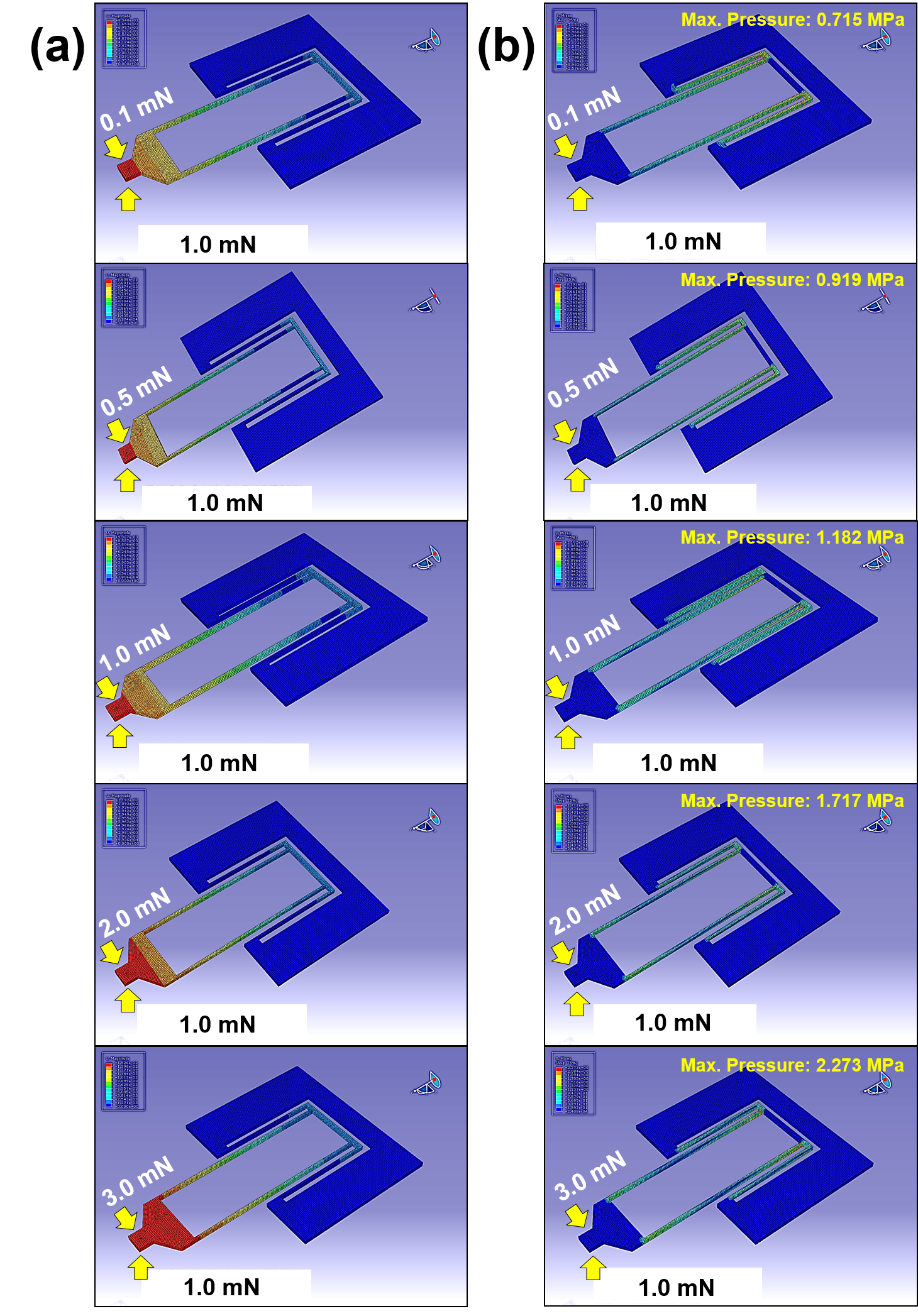

Supplement: Supplementary file 1 [file micromachines-10-00748-s001.zip › Figure S8.tiff]

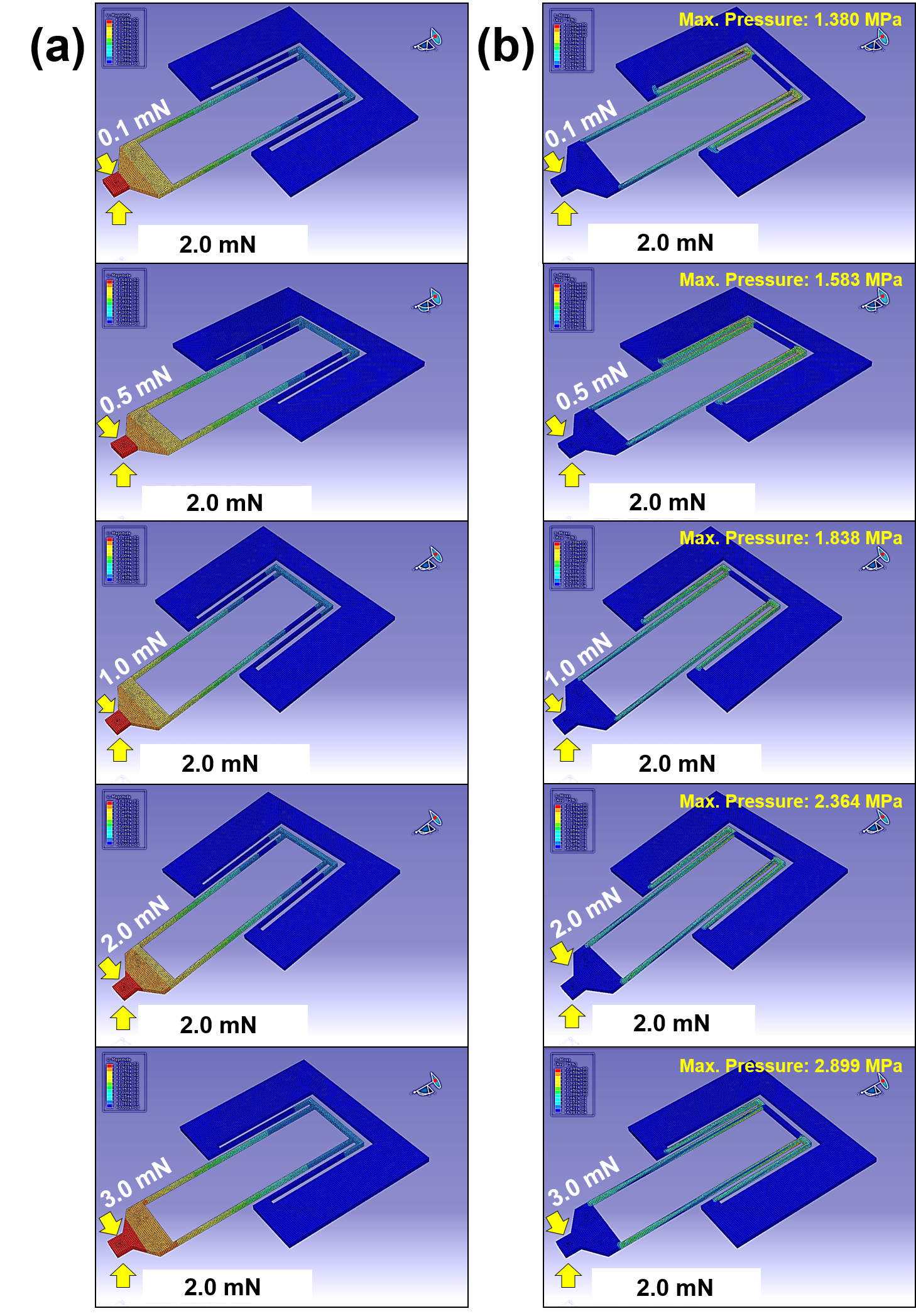

Supplement: Supplementary file 1 [file micromachines-10-00748-s001.zip › Figure S9.tiff]
